# Improving diet, activity and wellness in adults at risk of diabetes: randomized controlled trial

**DOI:** 10.1038/nutd.2016.42

**Published:** 2016-09-19

**Authors:** G Block, K M J Azar, R J Romanelli, T J Block, L P Palaniappan, M Dolginsky, C H Block

**Affiliations:** 1 Turnaround Health, Berkeley, CA, USA; 2 Sutter Health Research, Development and Dissemination, Walnut Creek, CA, USA; 3 Palo Alto Medical Foundation Research Institute, Palo Alto, CA, USA; 4 Stanford University School of Medicine, General Medical Disciplines, Stanford, CA, USA

## Abstract

**Objective:**

The purpose of this analysis is to examine the effect of an algorithm-driven online diabetes prevention program on changes in eating habits, physical activity and wellness/productivity factors.

**Methods:**

The intervention, Alive-PD, used small-step individually tailored goal setting and other features to promote changes in diet and physical activity. A 6-month randomized controlled trial was conducted among patients from a healthcare delivery system who had confirmed prediabetes (*n* =339). Change in weight and glycemic markers were measured in the clinic. Changes in physical activity, diet and wellness/productivity factors were self-reported. Mean age was 55 (s.d. 8.9) years, mean body mass index was 31 (s.d. 4.4) kg m^−2^, 68% were white and 69% were male.

**Results:**

The intervention group increased fruit/vegetable consumption by 3.71 (95% confidence interval (CI) 2.73, 4.70) times per week (effect size 0.62), and decreased refined carbohydrates by 3.77 (95% CI 3.10, 4.44) times per week both significantly (*P*<0.001) greater changes than in the control group. The intervention group also reported a significantly greater increase in physical activity than in the control group, effect size 0.49, *P*<0.001. In addition, the intervention group reported a significant increase in self-rated health, in confidence in ability to make dietary changes and in ability to accomplish tasks, and a decrease in fatigue, compared with the control group. These changes paralleled the significant treatment effects on glycemic markers and weight.

**Conclusions:**

In addition to promoting improvements in weight and glycemic markers, the Alive-PD program appears to improve eating habits and physical activity, behaviors important not just for diabetes prevention but for those with diagnosed diabetes or obesity. The improvements in wellness/productivity may derive from the diet and activity improvements, and from the satisfaction and self-efficacy of achieving goals.

## Introduction

In the United States, approximately two-thirds of adults are overweight or obese, almost 10% have diabetes and another one-third have prediabetes. Increased physical activity and improved dietary habits are the cornerstones of recommendations to prevent or manage these major health problems faced by developed nations. Yet despite national and disease-specific dietary and physical activity recommendations, both adults in general and persons with diabetes in particular appear to be falling far short in achieving these recommendations. In the United States, fewer than half of adults meet the national physical activity guidelines;^1^ fewer than 25% of adults meet the fruit and vegetable recommendation;^2^ and fewer than 40% of persons with diabetes meet the American Diabetes Association physical activity guidelines.^3^ Given the extent of these inadequate lifestyle behaviors in the United States and worldwide, cost-effective approaches are needed to help those with or at risk for diabetes and other chronic diseases to attain and sustain the behavioral changes needed to improve these conditions.

In a previous report we have demonstrated significant treatment effects of a fully automated online diabetes prevention program, Turnaround Health's Alive-PD, on body weight and biologic markers of diabetes risk (fasting glucose and hemoglobin A1c).^4^ The purpose of the present report is to analyze the effect of the intervention on changes in eating habits, physical activity and wellness/productivity variables.

## Materials and Methods

### The trial and primary outcomes

Alive-PD is a 1-year intervention designed to prevent the development of type 2 diabetes through improvements in eating habits and physical activity. The methods of the trial and the intervention program have been described in detail previously.^4, 5^ Briefly, Alive-PD provides individually tailored weekly small-step goal setting, tracking, a team system for social support and other activities. The weekly goals are derived from an extensive dietary and physical activity questionnaire completed at baseline and quarterly. The goals suggest small changes in frequency, portion size and type of foods reported by the participant, as well as gradual increases in physical activity.

The program promotes the following behaviors: decreases in simple sugars and refined carbohydrates; decreases in trans fats and saturated fat, if those are found to be excessive in the participant's diet; increases in fruit (excluding fruit juice) and vegetables (excluding potatoes); increases in legumes and nuts and seeds; and increase in aerobic activity, consistent with the US Physical Activity Guidelines for adults.

The program is delivered via web and email, supplemented by automated interactive voice response phone calls and a supportive smartphone application. All features of the program and contacts with participants are automated and algorithm-driven, without personal contact or coaching, either in-person or remotely.

The Alive-PD Study was a randomized, wait-list (usual care) controlled trial among 339 adults with clinically confirmed prediabetes. The study was approved by the Institutional Review Boards of the Palo Alto Medical Foundation and the Independent Review Board for Turnaround Health. Informed consent was obtained from all participants. The trial is listed on clinicaltrials.gov, NCT01479062. Participants were randomized by computer algorithm to begin the Alive-PD program immediately (intervention) or after 6 months (wait-list usual-care control). Participants were assessed in the clinic at baseline, 3 and 6 months. Clinic staff were blinded to randomization group.

Effects of Alive-PD on the primary end points of hemoglobin A1c (HbA1c), fasting glucose and weight have been reported previously.^4^ In intention-to-treat analysis, the Alive-PD group had both clinically and statistically significantly greater improvements than the control group, in HbA1c, fasting glucose, body weight, body mass index, waist circumference, triglyceride/high-density lipoprotein ratio and Framingham 8-year diabetes risk score. In addition, there were treatment effects among the small number of participants who had fasting glucose in the diabetes range at baseline.^4^

In the present analysis we report on changes in dietary behavior, physical activity and psychosocial factors that accompanied those physiologic changes.

### Questionnaire data

The detailed diet/activity questionnaire that provided the basis for tailored goals was only administered to the intervention group, so as not to sensitize the control group to desirable changes. As we wish, in this analysis, to compare diet/activity changes in control and intervention groups, results from that detailed questionnaire are not reported in this analysis and will be reported elsewhere.

Instead, here we report results from a few brief questions that were administered to both intervention and control, to permit comparison between the treatment groups. Intervention and control participants were asked five summary questions on their eating habits and one question on physical activity (Table 1). These questions are based on the summary questions used in the Block Questionnaire, which has been extensively validated.^6^ These six questions are the only items in which the intervention and control groups can be compared with respect to dietary and activity changes.

A number of personal wellness variables were asked about, and are grouped under the category ‘wellness/productivity'. They may be seen in the third section of Table 1, and include self-rated health status, self-efficacy for changing diet, difficulty concentrating at work and accomplishing tasks, and other factors. A fatigue quality-of-life indicator based on five sub-items was derived from Hartz *et al.*^7^ A stress indicator based on four sub-items was derived from Cohen *et al.*^8^ An immunity scale based on three sub-items was derived from Ware *et al.*^9^ These ‘wellness/productivity' variables may be thought of as potential mediators of the physiologic and glycemic outcomes, or as health-related quality-of-life indicators. Here we conceptualize them primarily as outcomes in themselves.

These questions were administered at baseline, 3 and 6 months. The diet and activity questions were administered online, while the wellness/productivity questions were administered in the clinic, although there was some overlap in the subject matter of the online and clinic-administered questions. Completion rates for the 6-month in-clinic questionnaire were 83% (136/163) by the intervention and 89% (156/176) by the control group. For the online questionnaire, completion rates were 62% (101/163) and 84% (147/176), respectively.

### Statistical methods

The sample was designed to provide 80% power to detect a minimum detectable difference in change in HbA1c of 0.48%. Treatment group differences in baseline characteristics were compared by *χ*^2^-tests for categorical variables and *t*-tests for continuous variables. Treatment group differences in outcomes were evaluated using linear regression approaches. In all models, change in the outcome of interest (for example, fruit consumption) was the dependent variable, with treatment group as the main predictor (independent) variable and baseline value of the outcome variable as a covariate. Variances were similar in the two treatment groups. We examined potential interactions with treatment group by variables that were expected *a priori* to be potential effect modifiers (sex, race/ethnicity, age and body mass index category) by inclusion of a cross-product term in the model. No significant interactions were found. Adjustment for age, sex, body mass index and race/ethnicity did not materially alter the results. For greater comprehensibility of graphs, the scoring of some variables was reversed, so that improvement was represented by a rising line.

Effect size was calculated as Hedges *g*. Hedges *g*, similar to Cohen's *d*, is the difference between the two mean changes (for intervention and control conditions, respectively) divided by the pooled s.d. In the Hedges *g* calculation, each group's s.d. is weighted by its sample size to calculate the pooled s.d.,^10^ making it more appropriate for use when the sample sizes of the two groups differ.

We performed analyses on the ‘completers' population, defined as those who completed the 6-month follow-up questionnaires, as well as the intention-to-treat population. All analyses were conducted using SAS software version 9.4 (copyright 2012, SAS Institute, Inc., Cary, NC, USA). For all tests of hypotheses, a two-sided *P*-value <0.05 was considered statistically significant.

## Results

### Sample characteristics

Participants (*n* =339) were randomized to intervention (*n* =163) who began the program immediately, or control (*n* =176) who began the program after 6 months. Participants ranged from 31 to 70 years of age, mean 55 (s.d. 8.9) years (Table 2). Men represented 69% (233/339) of the sample. In addition to the data in Table 2, 68.1% of the sample (231/339) had metabolic syndrome, 95% had prediabetes by fasting glucose, while 45% had prediabetes by HbA1c (data not shown).

At baseline, participants reported engaging in leisure-time aerobic activity on a mean of 2.29 (s.d. 1.89) days per week. They reported eating fruits and vegetables 1.28 (0.91) times per day, or about nine times per week.

### Changes in physical activity and food habits

**Figure 1:**
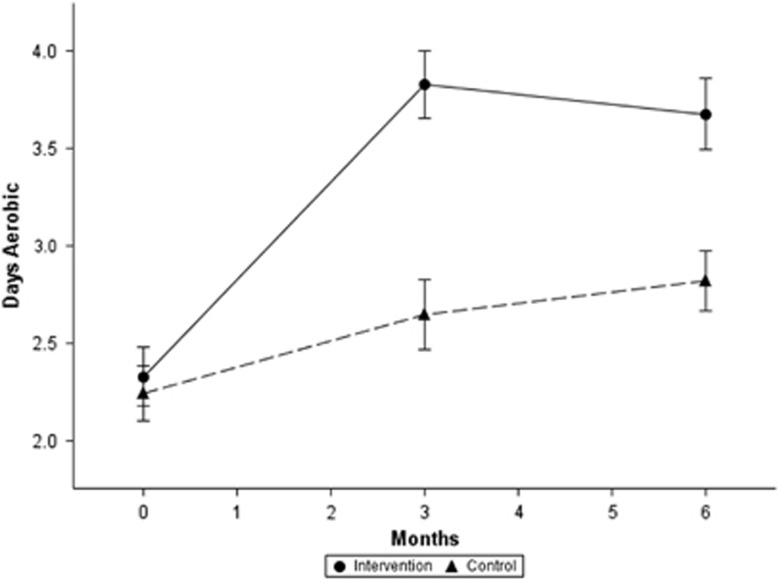
Changes in leisure-time aerobic activity. Solid line, intervention; dashed line, control. As measured by the ‘days aerobic' question in Table 1. Error bars: ±s.e.

**Figure 2:**
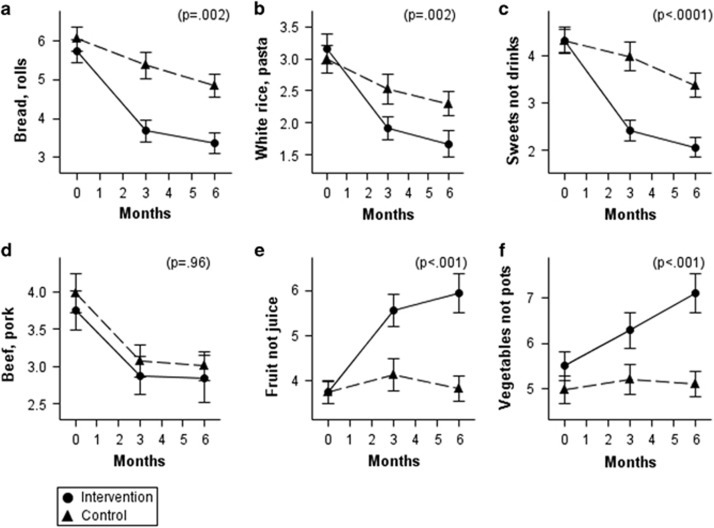
Changes in dietary factors. Solid lines, intervention; dashed lines, control. See Table 1 for exact wording and scoring of questions. (**a**) Bread, bagels and rolls. (**b**) White rice, spaghetti and pasta. (**c**) Pastries and sweets (not counting beverages). (**d**) Beef, pork and sausage. (**e**) Fruits (not counting juice). (**f**) Vegetables (not counting potatoes). Error bars: ±s.e. *P*-values: significance of treatment group difference at 6 months.

The patterns of changes in aerobic activity and food habits among completers over time are presented in Figures 1 and 2. Both the intervention and control groups changed their behaviors in physical activity (Figure 1) and some foods. Both groups declined in intake of bread/rolls, pasta/white rice and sweets (Figures 2a–c). Both groups declined in consumption of red meat (Figure 2d). (Red meat was not a focus of the Alive-PD intervention.) The intervention group significantly increased their intake of fruits and vegetables (Figures 2e and f), while the control group did not change their intake of those foods.

Effect sizes for changes in fruit and combined fruit and vegetable were substantial, 0.58 and 0.62, respectively, more than half a s.d. (Table 3). The effect sizes for aerobic activity, refined carbohydrates, sweets and vegetables were in the small-to-moderate range, 0.34–0.49 (one-third to one-half a s.d.). In addition, it is notable that for all food and activity variables except red meat, significant differences between intervention and control were also seen in intention-to-treat analysis (Table 3).

### Changes in wellness and productivity variables

Because the wellness/productivity variables are scales having no consistent or intrinsic meaning (unlike times per week, for example), their changes are reported here only in graphs comparing intervention and control group changes over time. The significance of the difference in changes between intervention and control was measured using a regression approach as described above.

**Figure 3:**
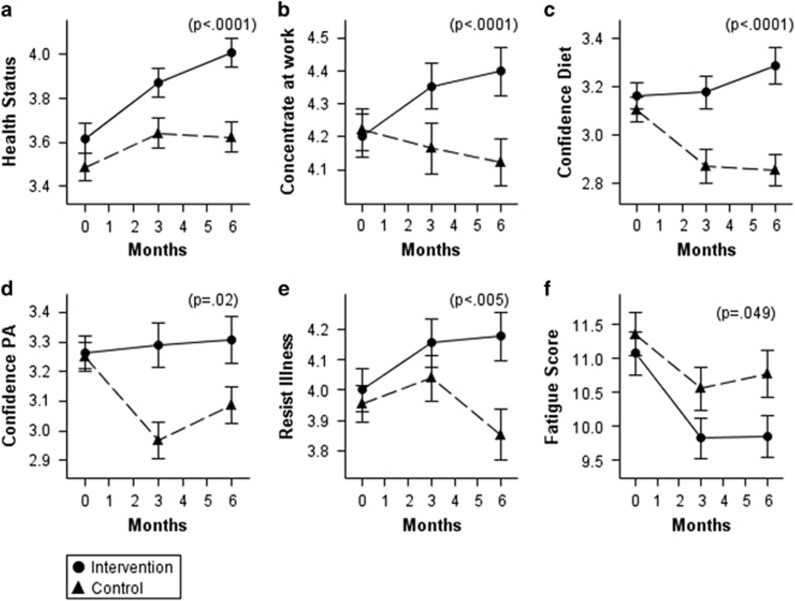
Changes in wellness/productivity questions. Solid lines, intervention; dashed lines, control. See online appendix for exact wording and scoring of questions. (**a**) Self-rated health status. (**b**) Difficulty concentrating/accomplishing at work. (**c**) Confidence in ability to change diet. (**d**) Confidence in ability to increase physical activity. (**e**) Body resists illness. (**f**) Fatigue score (Hartz *et al.*^7^). Error bars: ±s.e. *P*-values: significance of treatment group difference at 6.

The intervention group had significantly greater improvements than did the control group, for several important variables (Figure 3). These included self-rated health status, confidence in their ability to make lasting changes in diet and ability to concentrate and accomplish at work (all *P*<0.0001 for difference in change between intervention and control). Change in confidence in ability to make changes in physical activity was also significantly different between the two groups, *P*=0.02. In addition, the intervention group reported a significantly greater ability to resist illness, whereas the control group did not (*P*=0.005), and a significantly lower fatigue score (*P*=0.049).

The intervention group improved significantly more than the control group in two other wellness variables not shown in the graphs: energy level (*P*=0.01) and having a good appetite (*P*=0.01). In addition, the Ware Immunity Score improved more in the intervention than in the control group, but the difference in the changes did not reach statistical significance (*P*=0.07). Finally, changes in several wellness variables between treatment groups were not statistically significant, *P*>0.10: pain in the back, neck or shoulders; trouble sleeping; feeling depressed; and the Cohen stress score (data not shown).

## Discussion

### Diet and physical activity

In this randomized controlled trial of a fully automated diabetes prevention program, the intervention group showed significant improvements in physical activity and dietary variables compared with the control group through 6-month follow-up, all factors known to be important in weight management, diabetes prevention and behavioral management among persons with diabetes. These findings parallel previously reported findings from this trial on significantly greater reductions in weight and glycemic markers in the intervention compared with the control group.^4^

Although the diet and activity variables are self-reported, they are given a measure of credibility by the fact that significant changes were also seen in the relevant biological variables, HbA1c and fasting glucose. Moreover, the significant changes in diet and activity seen in the intervention group are consistent with our earlier work using a predecessor of the Alive-PD program, called Alive!^11^ In that study, we found significant increases in minutes of moderate and vigorous activity, as well as significant and positive changes in diet.

### Wellness/productivity effects

The wellness/productivity indicators seen in the intervention group are notable. Self-rated health status (Figure 3a) has been found repeatedly to predict future mortality,^12^ chronic disease incidence,^13^ diabetes incidence^13^ and diabetes mortality,^14^ and future medical care expenditures.^15^ The ‘ability to concentrate and accomplish work tasks' (Figure 3b) is key to productivity, and was also found in previous research to be significantly increased in the intervention group.^16^ Similarly, the reduction in the fatigue quality-of-life score is important for general wellness as well as productivity. The improvement in the ‘resist illness' question (Figure 3e) was unexpected. Such an improvement would be consistent with an increase in fruits and vegetables seen in the intervention group.

### Changes in the control group

The control group reported significant decreases in sweets and refined carbohydrates and significant increases in physical activity, in the present analysis. All participants were told on enrollment that they had prediabetes, and briefly told that weight loss and changes in physical activity and eating habits could reduce the risk of progression. We assume that this prompted efforts on the part of the control group to attempt change on their own. The control group did lose weight, decrease their intake of sweets, bread and other refined carbohydrates, and increase their physical activity somewhat. These are all recommendations for reducing diabetes risk that are either widely known or easily discovered. On the other hand, the control group did not increase their intake of fruits and vegetables (Figures 3e and f). Thus, it appears that some but not all of the recommendations for reduction in diabetes risk are known by many in the general public.

It also appears important that the control group reported a decrease in their confidence in their ability to make changes in diet and physical activity (Figures 3c and d), in contrast to the positive changes seen in the intervention group. Among the wellness/productivity indicators, the control group experienced negative although nonsignificant changes in ‘resist illness' and ‘concentrate at work', and increased only slightly and not significantly in self-rated health status.

It has been argued that control groups are not necessary in studies of interventions for diabetes prevention or weight loss because, it is argued, control groups do not change substantially.^17^ Our data conflict with this assumption. Our control group significantly decreased glycemic markers, weight and other hard end points, in intention-to-treat analysis.

### Existing guidelines

The US national guidelines for physical activity call for engaging in moderate or vigorous physical activity for 150 min a week.^18^ Physical activity recommendations for persons with prediabetes are similar to the national guidelines. For management of type 2 diabetes, the American Diabetes Association has recommended 150 min of moderate or vigorous activity per week, with at least 3 days per week of aerobic activity.^3, 19^

The national guidelines for dietary behavior^20^ have been broad and qualitative, calling for calorie balance, reductions in saturated and trans fats, reductions in added sugar and in foods that contain refined grains, and increase in consumption of fruits and vegetables. The most quantitative dietary recommendation is that from the US Department of Agriculture,^21^ which recommends that adults should eat 2–3 cups of vegetables and 1.5–2 cups of fruits per day.

Dietary recommendations for persons with diabetes and prediabetes have similarly been less quantitative than those for physical activity. The American Association of Diabetes Educators' practice guidelines call for helping patients to make healthy food choices, portion control, fat control and increasing intake of vegetables and fruits.^22^

### Previous research on improving diet and activity

Kohl *et al.*^23^ conducted a review of reviews or meta-analyses of studies of internet-delivered lifestyle interventions published between 2005 and 2012. Three reviews focused on interventions for physical activity in adults.^24, 25, 26^ Davies *et al.*^24^ found a small effect, with a weighted mean difference (Cohen's *d*) of 0.14. Van den Berg *et al.*^26^ identified three studies in adults, of which two found significant but small effects. Vandelanotte *et al.*^25^ identified four interventions for physical activity in adults, of which two had a statistically significant effect, with a median effect size of 0.36.

Kohl *et al.* found only three reviews of dietary interventions, of which two were in children or college students. No reviews reported interventions on sugar or refined carbohydrates, the subjects of three of our dietary variables. Harris *et al.*^27^ found 21 interventions designed to increase fruit and vegetable intake. They resulted in a weighted mean difference (Cohen's *d*) of 0.24 for fruits and vegetables.

Direct comparison of these studies with the Alive-PD results is difficult, as the metrics differ (for example, MET-minutes vs days per week). However, effect sizes reflect changes expressed in s.d.'s. The two effect sizes in previous studies^24, 25^ of physical activity, 0.14 and 0.36, are substantially smaller than the 0.49 seen for aerobic activity in the Alive-PD study. In terms of dietary changes, the effect size seen in Harris *et al.* of 0.24 for fruits and vegetables is considerably smaller than the effect size of 0.62 for fruits and vegetables in Alive-PD.

### Limitations

The fact that the diet and activity changes reported here are self-reported is a limitation. However, as noted, they are given some credibility by the parallel changes seen in the ‘hard' end points of HbA1c, fasting glucose and weight. The response rate to the online diet and activity questionnaire is a limitation (control 84% and intervention 62%), but it is notable that statistical significance is retained when the analysis is by intention to treat. Furthermore, the response rate seen for the wellness/productivity variables is 86%, increasing confidence in results for those variables.

### Future directions

Although these effects were achieved in the context of a diabetes prevention program, the Alive-PD program can be adapted for use in persons with other health conditions, such as obesity, metabolic syndrome and high cholesterol or hypertension. In addition, it could be used in persons with diagnosed diabetes, to complement diabetes self-management education (DSME) programs. Although the computer code underlying Alive-PD is not directly available, the program can be obtained through Turnaround Health, www.turnaroundhealth.com.

DSME has been shown to significantly reduce fasting glucose and HbA1c.^28^ However, Klein *et al.*^29^ noted that ‘it is critical that DSME programs increase their effectiveness, sustainability and scalability'. In their examination of 52 DSME programs with 9631 participants, Klein *et al.* found that only 7.23% of DSME participants moved to prediabetes or below. In the present study there were only 8 persons with diabetes, but the results are suggestive and call for further research: all 5 of those in the intervention group moved to prediabetes or normal, whereas none in the control group did so. Independent of the possibility that Alive-PD can help reduce glycemic markers in persons with diabetes, it could reinforce the behavioral messages of diabetes educators, maintain long-term contact or even reach some of the 50% of persons with diabetes who do not receive such education.^30^

## Conclusions

This analysis has found that in addition to effecting significant improvements in glycemic markers and body weight, the Alive-PD program produced improvements in physical activity and dietary factors that were statistically significantly greater than in the control group. In addition to diet and activity effects, participation in Alive-PD produced significant improvements in wellness and productivity variables, including self-rated health status. These reflect an important contribution of this program to quality-of-life issues.

As Alive-PD is delivered entirely ‘virtually', with no live coaches, it can be delivered widely and at low cost. The authors hope that it can be used to assist the approximately half of the US population who are in need of the glycemic and behavioral intervention it provides.

## Figures and Tables

**Table 1:** Questions and response categories

*Label*	*Questions*	*Response categories*	*Notes*
*Physical activity*			
Days aerobic	In your leisure time (when you are not working or doing household or family chores): how many days do you do an aerobic activity, such as brisk walking for fitness, exercise class and cardio machines such as exercise bike?	1: Rarely do it. 2: 1 day per week. 3: 2 days. 4: 3 days. 5: 4 days. 6: 5 days. 7: 6–7 days per week	a
			
*Food habits*			
Fruit	How often do you eat any fruit, not counting juice?	1: Once a week or less often. 2: 2–3 times a week. 3: 4–6 times a week. 4: Once a day. 5: 2 times or servings a day. 6: 3+ times or servings a day	b
Vegetables	Not counting potatoes, how many servings of vegetables do you eat, including in salad? Count a serving as 1 cup or about the size of your closed fist	1: Once a week or less often. 2: 2–3 times a week. 3: 4–6 times a week. 4: Once a day. 5: 2 times or servings a day. 6: 3+ times or servings a day	b
Bread	How often do you eat any bread, bagels, rolls and so on?	1: Less than once a week. 2: Once a week. 3: 2–3 times a week. 4: 4–6 times a week. 5: Once a day. 6: 2+ a day	b
White rice, pasta	How often do you eat any noodles, spaghetti, pasta and white rice?	1: Less than once a week. 2: Once a week. 3: 2–3 times a week. 4: 4–6 times a week. 5: Once a day. 6: 2+ a day	b
Refined carb	Sum of bread, white rice and pasta	Sum of times-per-week of bread and white rice, and pasta	
Red meat	How often do you eat any beef, pork, hamburger, ham and sausage?	1: Less than once a week. 2: Once a week. 3: 2–3 times a week. 4: 4–6 times a week. 5: Once a day. 6: 2+ a day	b
Sweets	How often do you eat any sweets or pastry, such as cookies, cake, ice cream and candy?	1: Less than once a week. 2: Once a week. 3: 2–3 times a week. 4: 4–6 times a week. 5: Once a day. 6: 2+ a day	b
			
*Wellness/productivity*			
Health status	During the past 3 months, how would you rate your general health?	1: Poor. 2: Fair. 3: Good. 4: Very good. 5: Excellent	c
Concentrate at Work	During the past 4 weeks, how much difficulty did you have concentrating at work or accomplishing tasks because of physical or emotional problems?	1: Could not do daily activities. 2: Quite a lot. 3: Somewhat. 4: Very little. 5: Not at all	c
Confidence diet	How confident are you that you can make or maintain lasting changes to reduce sweets and saturated fat?	1: Not at all. 2: Might be able to. 3: Pretty sure I can. 4: Very confident	
Confidence Physical activity	How confident are you that you can make or maintain lasting changes to be more physically active	1: Not at all. 2: Might be able to. 3: Pretty sure I can. 4: Very confident	
Resist illness	My body seems to resist illness very well	1: Definitely false. 2: Mostly false. 3: Don't know. 4: Mostly true. 5: Definitely true	c
Fatigue score	Sum of five sub-items (low energy, tired, woke up fresh, fatigue interfered and fatigue was disabling)	1: None of the time. 2: A little of the time. 3: Some of the time. 4: A good bit of the time. 5: Most of the time	d
Energy	During the past 4 weeks, how much were you bothered by: feeling tired or having low energy	1: Not at all. 2: Very little. 3: Somewhat. 4: Quite a lot. 5: Very much	
Immunity score	Sum of three sub-items (get sick easier, catch what is going around and I resist illness)	1: Definitely false. 2: Mostly false. 3: Don't know. 4: Mostly true. 5: Definitely true	e
Pain	During the past 4 weeks, how much were you bothered by pain in your back, neck or shoulders	1: Not at all. 2:Very little. 3: Somewhat. 4: Quite a lot. 5: Very much	
Sleep	During the past 4 weeks, how much were you bothered by trouble sleeping	1: Not at all. 2:Very little. 3: Somewhat. 4: Quite a lot. 5: Very much	
Depression	How often do you feel depressed, sad, blue, hopeless	1: Almost never. 2: Sometimes. 3: Quite often. 4: Very often.	
Stress score	Sum of four sub-items (unable to control, can handle, optimistic and difficulties piling up)	1: Never. 2: Almost never. 3: Sometimes. 4: Fairly often. 5: Very often	f

a Converted to days per week.

b Converted to times per week.

c Originally asked in opposite direction, reversed here for clarity of graphs.

d Calculated per Hartz *et al.*^7^

e Calculated per Ware.^9^

f Calculated per Cohen and Williamson.^8^

**Table 2:** Participant characteristics

*Variable*^a^	*Category*	*All*	*Intervention*	*Control*	P^b^
		N= *339*	N= *163*	N= *176*	
Age, years		55.0±8.9	55.0 (8.8)	54.9 (9.1)	0.88
Female, *n* (%)		106 (31.3)	52 (31.9)	54 (30.7)	0.81
College or above, *n* (%)		281 (82.9)	137 (84.1)	144 (81.8)	0.59
Race/ethnicity, *n* (%)^c^					0.07
	White	229 (67.6)	109 (66.9)	120 (68.2)	
	Hispanic	21 (6.2)	7 (4.3)	14 (8.0)	
	Asian	70 (20.7)	41 (58.6)	29 (41.4)	
	Other	19 (5.6)	6 (3.7)	13 (7.4)	
Weight, kg		92.9 (15.8)	93.7 (14.9)	93.3 (16.6)	0.68
BMI, kg m^−2^		31.2 (4.4)	31.1 (4.5)	31.2 (4.3)	0.73
Bread^c^, per week		5.90 (3.99)	5.74 (3.94)	6.06 (4.04)	0.46
Pasta, white rice, per week		3.07 (2.89)	3.16 (2.97)	2.99 (3.81)	0.60
Sum of bread, pasta, white rice, per week		8.97 (5.01)	8.89 (5.10)	9.05 (4.03)	0.78
Red meat, per week		3.87 (3.43)	3.75 (3.39)	3.98 (4.47)	0.53
Sweets, per week		4.31 (3.42)	4.30 (3.07)	4.31 (3.72)	0.96
Fruit, per week		3.74 (3.31)	3.75 (3.29)	3.74 (3.35)	0.98
Vegetables, per week		5.23 (4.01)	5.50 (3.99)	4.98 (4.03)	0.23
Sum of fruit and vegetables, per week		8.98 (6.39)	9.25 (6.25)	8.72 (6.52)	0.44
Aerobic activity (days per week)		2.29 (1.89)	2.33 (1.92)	2.25 (1.87)	0.68

Abbreviation: BMI, body mass index.

a Mean (s.d.) unless otherwise indicated.

b Significance of difference between intervention and control.

c Race and ethnicity as reported on online questionnaire. Native American/Alaskan, Native Hawaiian/Pacific Islander, more than one race or ‘not reported' reported as ‘other'.

**Table 3:** Mean baseline-adjusted change in intervention and control groups, effect size and significance of treatment effect in completers and intention-to-treat population

*Variable (per week)*	*Change in completers at 6 months (95% CI)*				
	*Alive-PD* n=*100*^a^	*Control* n=*147*^a^	*Effect size*^b^	P^c^	P^d^
Aerobic activity (days per week)	1.21 (0.94, 1.47)	0.42 (0.20, 0.64)	0.49	<0.001	<0.001
Red meat	−0.91 (−1.31, −0.51)	−0.93 (−0.26, −0.60)	0.07	0.95	0.52
Bread	−2.39 (−2.91, −1.87)	−1.29, (−1.72, −0.86)	0.19	0.002	0.046
Pasta, white rice	−1.40 (−1.32, −0.78)	−0.69 (−0.83, −0.33)	0.36	0.001	0.010
Sum of bread, pasta, white rice	−3.77 (−4.44, −3.10)	−1.99 (−2.55, −1.44)	0.34	<0.001	0.006
Sweets	−2.26 (−2.69, −1.82)	−1.02 (−1.38, −0.67)	0.40	<0.001	0.011
Fruits	2.03 (1.43, 2.62)	0.09 (−0.41, 0.58)	0.58	<0.001	<0.001
Vegetables	1.75 (1.14, 2.35)	0.05 (−0.45, 0.55)	0.43	<0.001	<0.001
Sum of fruits and vegetables	3.71 (2.73, 4.70)	0.16 (−0.65, 0.98)	0.62	<0.001	<0.001

Abbreviation: CI, confidence interval.

a 6-month value and 95% confidence limits from least squares means in models of following form: 6-month value=baseline+treatment group.

b Hedges *g*. Similar to Cohen's *d*, Hedges *g* is the difference between the two mean changes divided by the pooled s.d., and represents the difference in s.d.'s.

c Significance of the difference in change in intervention vs control groups over 6 months, from model described in ‘a'.

d Significance of the difference in change in intervention vs control groups over 6 months, from model described in ‘a': intention-to-treat analysis, all initially enrolled subjects, missing data imputed using Last Observation Carried Forward imputation approach.
